# Methods to explore productive behaviors in personal and extrapersonal space

**DOI:** 10.3389/fnhum.2014.00790

**Published:** 2014-10-02

**Authors:** Anna Sedda, Martina Gandola

**Affiliations:** Department of Behavioral and Brain Sciences, University of PaviaPavia, Italy

**Keywords:** perseveration, caloric vestibular stimulation, arm position sense, bimanual coupling, Right brain damage

Among other crucial functions such as emotional and numeric processing, the right hemisphere processes properties of space (Bartolomeo, 2006), of the body and of the sensory information related to it (Berlucchi and Aglioti, 2010). Being able to control the position of our arms and to coordinate our hands allows to perform reaching movements, precision grips, and other actions essential to interact with targets in the environments (Milner and Goodale, 1993). Sensory information from the vestibular system allows to balance body parts according to the state of the environment (Ferrè et al., 2013a). Finally, repeating an action only once if the first shot is successful allows to save precious energy that can be employed to perform other tasks (Miller and Durst, 2014). When a brain lesion occurs, all these abilities might be impaired, impacting the patient's ability to comply with everyday life requests (Sedda and Scarpina, 2012).

Importantly, the goal to understand the mechanisms behind these deficits cannot be reached unless a parallel path focuses on the development of reliable paradigms to explore them. Consequently, the standardization of methods to analyze the same phenomenon in different patients is of undeniable value, as well as the possibility to develop paradigms suitable to be applied in both patients and healthy subjects.

Such is the case of a particular form of perseveration in which patients produce continuous and uninterrupted movements (called “scribbles perseveration” in Gandola et al., 2013; Toraldo, 2013). Both, in case of visuo-spatial as well as in other diseases (such as Alzheimer dementia; Miozzo et al., 2013) the debate is still open on whether perseveration is a consequence of the pathology itself or it is only associated to it accidentally (Gandola et al., 2013). The method to calculate a Scribble Perseveration Index (SPI) developed by Toraldo (2013) could be virtually applied to all these diseases, to clarify this phenomenon even retrospectively. The adoption of the same method would allow a more precise comparison across pathologies, and consequently more reliable results. Further, previously acquired data could be re-read in light of this method and homogeneous results could be obtained.

Another example is represented by the study of Ferrè et al. (2013b), exploring vestibular modulation of spatial perception in healthy subjects by means of galvanic vestibular stimulation (GVS) and a line bisection task performed in near and far space (Ferrè et al., 2013b). Among the results, the authors highlight a shift in the line bisection bias from left to right as the distance that is being viewed increases, occurring independently from GVS. This finding is extremely interesting as it questions whether this vestibular modulation could be equally effective on neglect behaviors in the far and in the near space (Van der Stoep et al., 2013). This last study in particular is an example of the need to explore techniques that are already known to be effective in patients (Bottini et al., 2013) also in healthy subjects, as previously ignored phenomena could lead to new rehabilitative paths and ideas.

One challenge for experimental research on brain-damaged patients is to shift from qualitative or descriptive methodologies to more objective and experimental methods.

For instance, in the field of anosognosia for hemiplegia, pivotal reports were based on single cases descriptions (see review in Vallar and Ronchi, 2006). Recently, a profound methodological change has occurred and clinical descriptions have been replaced by well-controlled experiments, aimed to test specific models and hypotheses (Fotopoulou et al., 2008; Jenkinson et al., 2009; Garbarini et al., 2012; Pia et al., 2013). An example of this new approach is the use of the bimanual coupling paradigm to explore productive symptoms such as the false experience of movement in patients with anosognosia or delusion of ownership (Garbarini et al., 2012; Pia et al., 2013). The novelty of this simple yet brilliant methodology, as exposed in the review of Garbarini and Pia (2013), lies in the possibility to *objectively* measure the consequences of the presence of anosognosia for left hemiplegia (the belief of being able to move the paralyzed limb) on the motor performance of the healthy limb. The verbal delusion is explored indirectly through the effects that it produces on a measurable behavioral performance. By measuring delusional behaviors of brain-damaged patients and normal behaviors of healthy subjects with the same paradigm, it would be possible to obtain reliable and generalizable information on the underpinning cognitive processes that regulate the intention to move, motor programming and awareness. Remarkably, the possibility to apply the bimanual coupling paradigm also in patients with different delusion of ownership (e.g., patients that incorporate the examiner's hand in their body schema as described by Garbarini and Pia Garbarini et al., 2013 or patients with somatoparaphrenia) offers the possibility to measure and quantify the impact of these sometimes-elusive verbal delusions on motor performance.

Similarly, the standardization on healthy volunteers of a task apt to measure position sense by measuring angular deviations from a visually defined target separately for each arm as presented by Schmidt et al. (2013) allows to obtain objective data from patients suffering from disorders of arm position sense that often occur after unilateral stroke (Schmidt et al., 2013).

Both these tasks, bimanual coupling and arm position paradigms, are also important for other, not negligible reasons: firstly, as anosognosia for hemiplegia and disorders of arm position sense often present when patients are just a few days after a cerebral stroke (Vocat et al., 2010), they are not able to undergo complex tasks that requires a huge cognitive load. The simplicity of these tasks makes them appropriate to be used also in the acute phase after stroke. Secondly, these tasks can be easily adopted in clinical settings, such as stroke units, where sophisticated equipment for the recording of physiological parameters (i.e., those required to measure kinematics) are often not available.

Finally, putting together information from these tasks would contribute in producing a general model on arm control (both in terms of motor abilities and proprioceptive information) that would prove very useful in other fields, such as to develop neutrally controlled robotic arms that mimic biological limbs functioning (Andersen et al., 2004).

In conclusion, within this research topic we had the possibility to witness the importance to evaluate diverse paradigms and methodologies in order to understand impairments related to brain damage. Furthermore, these works have the merit to take into consideration the practical obstacles related to transferring experimental settings to clinical practices and they propose simple but efficient methods to overcome these obstacles.

## Conflict of interest statement

The authors declare that the research was conducted in the absence of any commercial or financial relationships that could be construed as a potential conflict of interest.
